# Risk of shoreline hardening and associated beach loss peaks before mid-century: Oʻahu, Hawaiʻi

**DOI:** 10.1038/s41598-020-70577-y

**Published:** 2020-08-12

**Authors:** Kammie-Dominique Tavares, Charles H. Fletcher, Tiffany R. Anderson

**Affiliations:** Department of Earth Sciences, School of Ocean and Earth Science and Technology, University of Hawaiʻi at Mānoa, 1680 East-West Rd., POST Room 721, Honolulu, HI 96822 USA

**Keywords:** Projection and prediction, Environmental impact

## Abstract

Shoreline hardening, which causes beach loss globally, will accelerate with sea level rise (SLR), causing more beach loss if management practices are not changed. To improve beach conservation efforts, current and future shoreline hardening patterns on sandy beaches need deeper analysis. A shoreline change model driven by incremental SLR (0.25, 0.46, 0.74 m) is used to simulate future changes in the position of an administrative hazard zone, as a proxy for risk of hardening at all sandy beaches on the island of O‘ahu, Hawai ‘i. In Hawai ‘i, hardening can be triggered when evidence of erosion is within 6.1 m (“20 ft”) of certain structures, allowing an applicant to request emergency protection. Results show an increase in shoreline vulnerability to hardening with SLR governed by backshore land use patterns. The largest increase (+ 7.6%) occurred between modern-day and 0.25 m of SLR (very likely by year 2050) with half of all beachfront shoreline at risk by 0.74 m of SLR. Maximum risk of shoreline hardening and beach loss is projected to occur from modern-day and near-term hardening because of the heavily developed aspect of some shoreline segments. Adaptation to SLR should be considered an immediate need—not solely a future issue.

## Introduction

**Figure 1:**
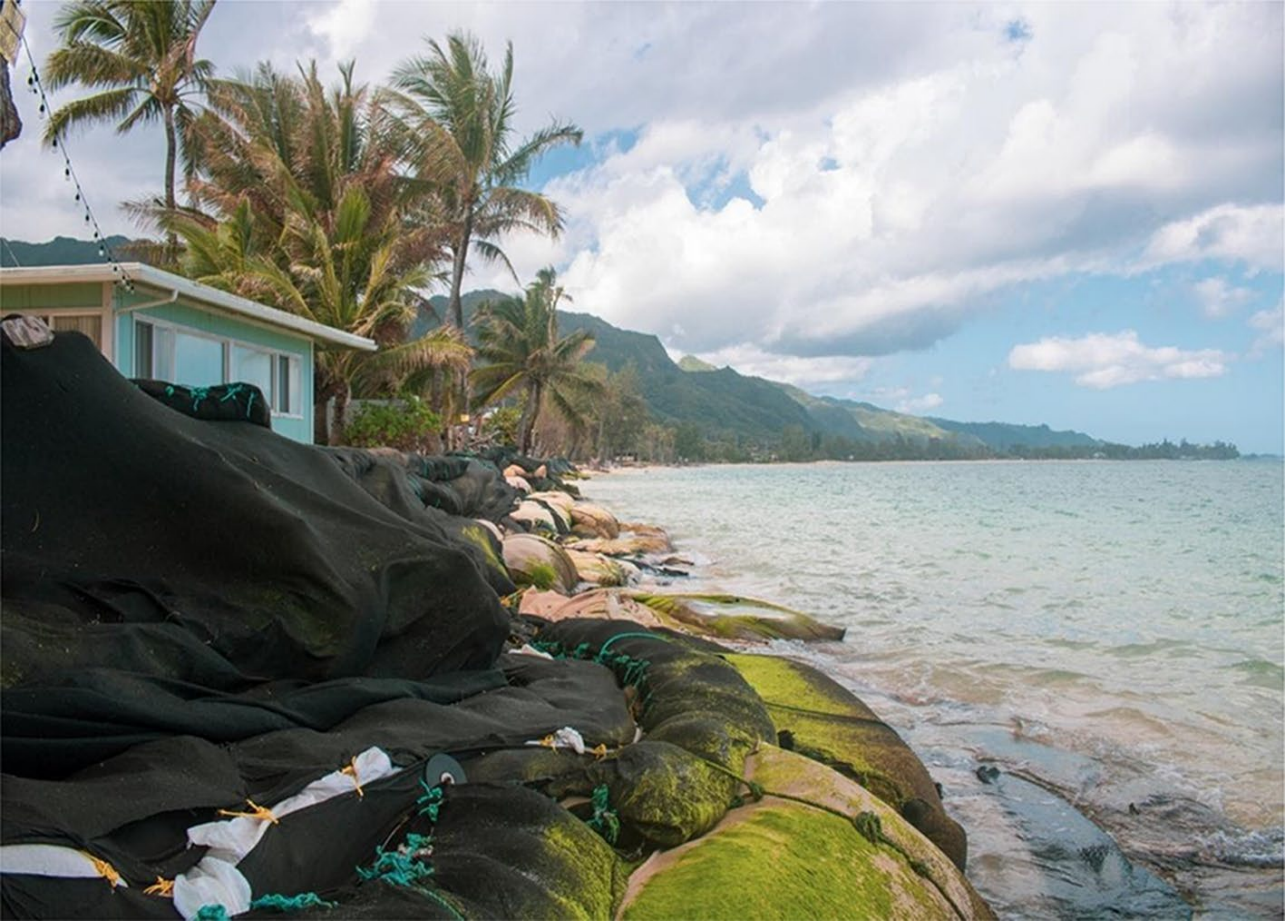
Hardening to protect backshore assets has resulted in widespread beach loss in Hawaiʻi. Photo of Koʻolauloa shoreline in March 2020 provided by contributing author Kammie-Dominique Tavares.

Beaches are critical ecosystems^1^, storm buffers^2^, an essential cultural setting^3^, and an attraction for tourists^4^. However, poor management of shoreline hardening, the construction of hard structures such as seawalls along the shore, has led to beach narrowing and loss around the world^5–8^ (Fig. 1). Hardening the shoreline prevents property erosion and protects backshore development from erosional hazards. However, it causes “coastal squeeze”^9^ or beach narrowing and eventual loss on chronically retreating shorelines^10^, an inevitability with sea level rise (SLR)^10–12^. This study analyzes current and future shoreline hardening patterns with SLR to improve the understanding of future challenges related to beach conservation.

Shoreline erosion is projected to occur along more of the shoreline as well as accelerate rates of erosion due to SLR^13–15^, increasing the length of shoreline at risk of hardening and beach loss if poor management practices continue. By the end of the century, SLR may cause half of the sandy beaches globally to disappear driven by shoreline dynamics and coastal recession alone^15^. With dense populations living near the coast now^16^ and likely in the future^17^, pressure to continue development alongshore and to protect existing development from rising sea level may lead to more shoreline hardening, and by extension beach loss. Gittman et al.^18^ estimated 14% of the US coastline has been hardened with strong correlation to population density, predicting one third of the shoreline could be hardened by 2100 if coastal populations continue to increase and the shoreline hardening rates stay the same (+200 km/year). Current and future potential hardening patterns need to be considered when managing sandy shorelines, especially under the context of SLR, because of its degrading effect on the health of sandy beaches^6^.

While much research has been done to understand the effect of shoreline hardening around the world (China^19^, Southern Europe^20^, Portugal^21^, United States^1^, Puerto Rico^22^, for example), research focused on the potential drivers for current and future hardening is limited. Gittman et al.^18^ compared hardening patterns to housing density, gross domestic product, coastal slope, accretion/erosion rates, geomorphology, mean tidal range, mean wave height, relative SLR, storm frequency, shoreline positions, and years since shoreline hardening was banned in that area for the coastline in the continental US. They offered future predictions of shoreline hardening development based on the understanding of the parameters described above. However, the effect of accelerated SLR was not considered. Others explore the effect of accelerated SLR on coastal assets, however, do not go into depth to understand current and future shoreline hardening patterns^12,23^.

**Figure 2:**
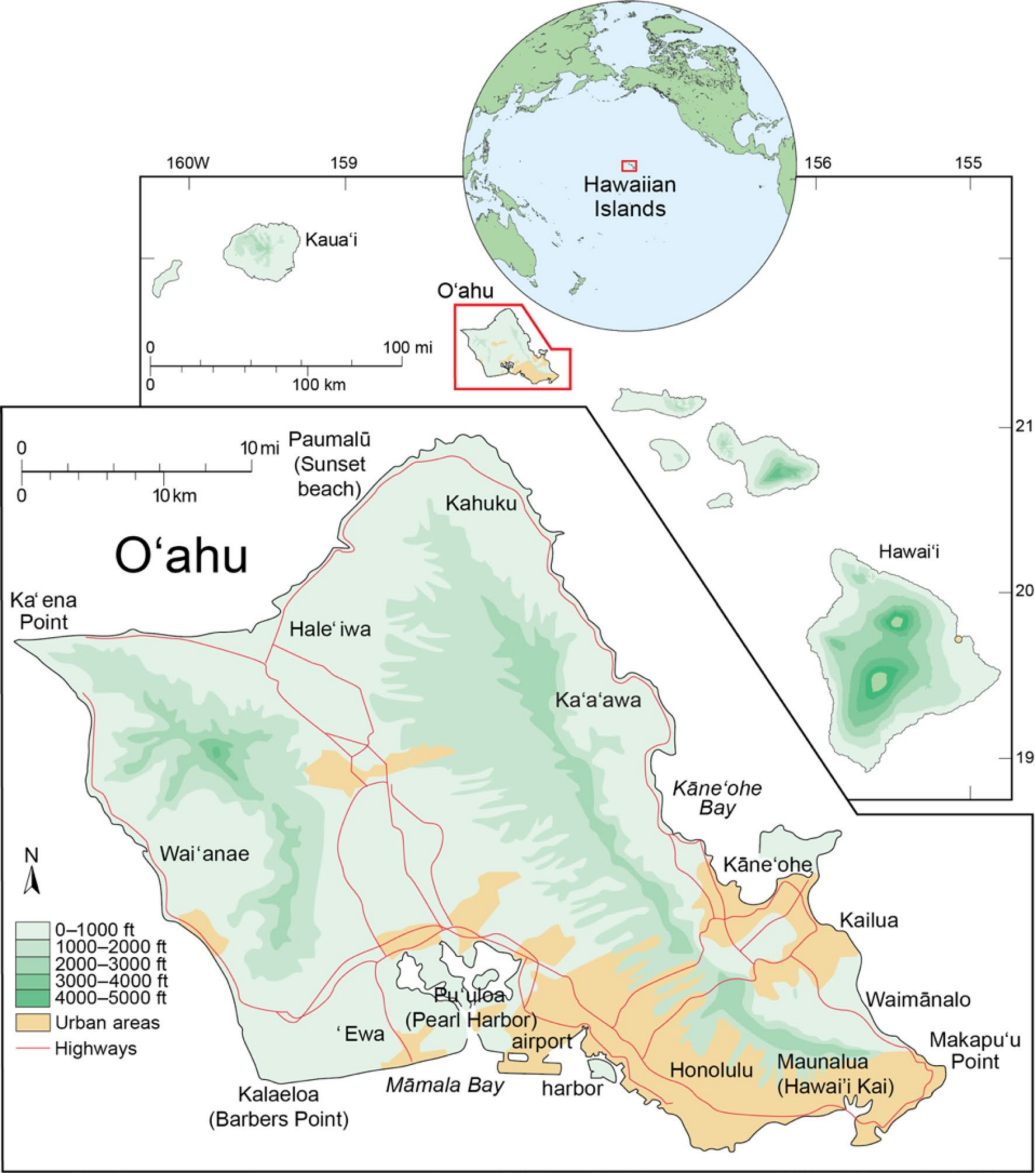
The island of Oʻahu, Hawaiʻi, has four fundamental shoreline orientations, north, east, south and west. Source: Fletcher, Mullane, and Richmond (1988). Reproduced with permission from the Coastal Education and Research Foundation, Inc.

To further this line of research and provide coastal managers and stakeholders with improved understanding of the impacts of shoreline hardening related to accelerated SLR, a chronology of threatened beach resources is established in combination with an analysis of backshore land use. In this study, local shoreline hardening management practices are applied to a shoreline change model that projects future erosion and shoreline retreat to create administrative erosion hazard zones for all historically sandy shorelines on the Hawaiian island of Oʻahu (Fig. 2). Categorized by land use, parcels hardened and at risk of hardening (within the administrative erosion hazard zones) were identified using shoreline change projections under three SLR scenarios (0.25, 0.46, and 0.74 m). Oʻahu is used as a living laboratory of land use decisions, and, by extension, these results are indicative of beach conservation stresses in similar situations around the world.

### Study site

Located in the North-Central Pacific, the island of Oʻahu (Fig. 2) is a volcanic island characterized by a fringing reef that creates alongshore variability, producing embayment and headland coastlines that are both carbonate and igneous in origin^24^. Beach development is dominated by longshore transport^25^ with overall historical shoreline change rates generally stable to accreting in embayed beaches and eroding in headland areas^26^. The backshore consists of a mixture of carbonate, marine and aeolian sediments produced from the withdraw of the Kapapa High Stand (3500–5000 year B.P.) and subsequent dune formation^27^.

Oʻahu is exposed to a highly variable and diverse wind-wave climate^28^. As a result, shoreline orientation is a fundamental parameter affecting beach characteristics. Oʻahu can be characterized by four principal orientations (north, east, south, and west). The north shore of Oʻahu experiences large winter swells generated by low pressure centers in the North Pacific. The west shore is characterized by both refracted winter and summer swells. The south shore of Oʻahu, exposed to summer swell and oblique trade wind waves, is the most heavily developed coastline on the island. The east side of the island experiences trade wind waves and, at its northernmost and southernmost ends, seasonally refracted swell. Projected shoreline changes island wide as well as for each of these directional segments are characterized.

### Beach management on Oʻahu, Hawaiʻi

In the U.S., partnerships between federal and local governments established management of coastal resources through the National Coastal Zone Management Program (NCZMP) under the National Oceanic and Atmospheric Administration. Based on NCZMP criteria, states design their own programs and set their own objectives. In Hawaiʻi, the national and local partnership is based on Hawaiʻi Revised Statutes §§ 205A^29^, the state Coastal Zone Management law, which declares protection of view planes, public access to and along the shoreline, and conservation of coastal ecosystems, especially beaches, as the primary purpose of beach management efforts. Summers et al.^5^ established that despite Hawaiʻi’s robust legal and planning framework, there has been a failure to achieve stated goals, especially due to poor management of shoreline hardening.

With a population of nearly 1 million people^30^, heavy development of homes, hotels, roads, and beach parks has occurred along Oʻahu’s shoreline and has infringed upon most beach dunes, leaving few natural dune systems in place^31^. All of this has subsequently been developed in the modern day CZM program with a statewide setback of 40 ft from the highest wash of the waves^29^. Development was managed under the assumption that sea level was not rising and that the shoreline was stable within an envelope of variability. However, property erosion soon proved otherwise, and shorelines fronting areas threatened by erosion were hardened to protect coastal development^32^. As a result of shoreline hardening, at least 8.7 km of beach has been lost on Oʻahu and 21.5 km statewide^33^.

For purposes of management, the State of Hawaiʻi defines an administrative shoreline, which is determined by evidence of the highest wash of the waves at high tide during the time of the year when the waves are highest^29^. The state uses this shoreline to identify cases under its jurisdiction where homeowners apply for emergency permits, often to harden the shoreline. Today, shoreline hardening typically occurs through emergency permits for coastal erosion hazards. This may also be the case in other areas where shoreline hardening is presently limited^20^*.* The criteria under which an emergency permit may be submitted is when “an inhabited dwelling, essential cultural or natural resource, or other (non-movable) major structure or public facility” falls within 20 ft (6.1 m) of evidence of active erosion^34^. In this paper, the 20 ft buffer is used to create an administrative erosion hazard zone in a GIS analysis as a proxy for future risk of hardening under three SLR scenarios on the beaches of Oʻahu.

## Methodology

**Figure 3:**
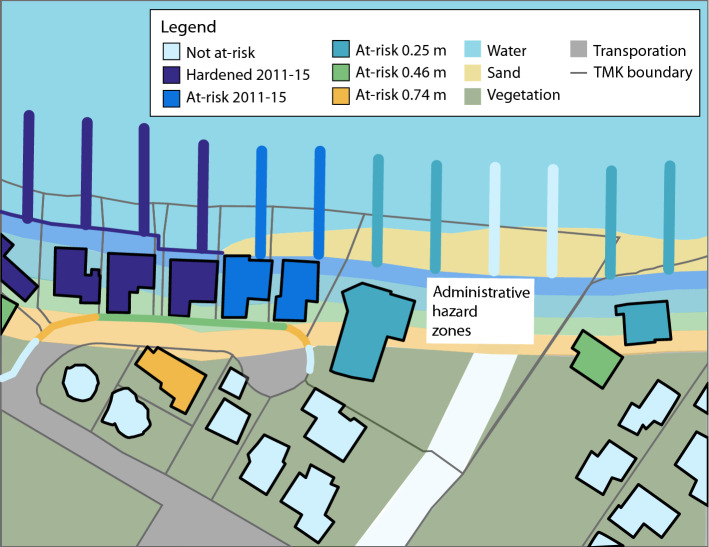
Conceptual diagram of shoreline development and structures at risk of hardening in modern-day (2011–15) and future erosion hazard zones (0.25, 0.46, and 0.74 m of sea level rise).

The greatest threat to beach conservation under conditions of accelerating SLR is shoreline hardening. To provide managers with improved understanding of this problem, a three-step process was used: (1) projecting future shoreline position and rate of change under three SLR scenarios using the probabilistic method described in Anderson et al.^13^, (2) identifying backshore parcels and their development pattern where an administrative trigger for hardening is crossed (20 ft buffer); and 3) defining land use categories that have historically been most likely to be hardened (Fig. 3).

### Future shoreline projection

Many models used to project shoreline change resulting from SLR fail to include historical data and, therefore, do not provide results reflecting site specific parameters^35^. Other models use projections of historical change without taking into account the accelerating nature of global mean SLR^36^. In this paper, the method described in Anderson et al.^13^ is used because it provides an approach that accommodates both of these needs by combining historical patterns of beach change with a geometric equilibrium profile model to identify shoreline retreat in response to SLR. Because the shoreline of O‘ahu (Fig. 2) has been carefully mapped for historical rates of change^33^, the method described in Anderson et al.^13^ offers an ideal opportunity to analyze projections of future shoreline change due to SLR and patterns of intersection with a database of backshore land use.

Using this method^13^, future shoreline positions are projected under three scenarios of SLR (0.25, 0.46, and 0.74 m). The SLR scenarios refer to the median probability values in the IPCC AR5 report^37^, which correspond to the years 2050, 2075, and 2100 respectively for Representative Concentration Pathway (RCP) 8.5. The RCP 8.5 “business as usual” greenhouse gas concentration pathway was used in this study as results are intended to inform the development of land use policies in a low risk tolerance framework.

The shoreline change model combines the long-term historical shoreline change rate^33^ with the conceptual geometric Bruun-type model of beach profile adjustment to SLR by Davidson-Arnott^38^, to project future shoreline positions and rates of change under each scenario. The projected amount of net shoreline change is characterized by a joint probability density function (pdf) that includes uncertainties arising from the historical shoreline change methodology^33^, the geometric model^38^, and the IPCC AR5 sea level projections^37^. From this pdf, the shoreline change that corresponds to the mode (or maximum probability density) is used to represent the most likely amount of projected net shoreline change in each scenario. (More details on the method and data used are available in the Supplementary Methods.) It is important to note that, while the method does capture spatially varying sediment behavior, this type of simplified approach cannot account for complex nonlinearities in sediment supply related to local geologic constraints and local variations in the rate of SLR. The reader is directed to the original method publication^13^ for a robust discussion of limitations to this approach.

The current vegetation line is used to represent the present-day administrative shoreline, as it is a reasonable approximation of the state’s shoreline definition, which describes the highest wash of the waves at high tide during the time of the year when the waves are highest^29^. The vegetation line is not only an administrative feature but also represents the long-term landward edge of the active beach for its account of seaward and landward shifts as well as the seaward boundary of backshore land. The modeled net future shoreline change is projected from the modern vegetation line under each scenario and is referred to as a projected shoreline.

### Shoreline hardening potential

Using GIS tools, the 20 ft buffer extending inland from each projected shoreline, referred to as the *administrative erosion hazard zone* in this paper, is identified and noted where it intersects habitable structures, transportation infrastructure, and public facilities (Fig. 3). Erosional threats to these three assets have historically been the frequent trigger for hardening^5^. The roofs of buildings were used to identify dwellings and public facilities. Transportation features were digitized on the most shore-parallel seaward edge.

Using GIS layers available online through the Hawaiʻi Office of Planning’s GIS Program^39^ (see Supplementary Table S1), all backshore land use on the island of Oʻahu was classified into four major categories: residential, city and state beach parks, federal lands, and unclassified lands (Table 1). While land use patterns may change over time, no change is assumed since predicting future land use is not possible.

**Table 1:** Definition of land use categories and patterns of hardening response based on observed modern-day hardening within each land use category.

	Residential	Beach park	Federal	Unclassified
Definition	TMK parcel with dwellings as well as golf courses	State or city beach park	Military and conservation land	Unclassified shoreline, typically roads or undeveloped land
Hardening to protect buildings	Hardening along the entire length of the parcel (up to 100 m on large parcels)	Hardening only in front of each threatened building, except for clusters of cabins, in which shoreline hardening spans the length of clustered cabins (Hardening typically spans 60–120 m of shoreline in these categories)		No buildings were in this land use
Hardening to protect transportation assets	Hardening only in front of the threatened portion of roadway or other transportation asset; except for cases where the greens of a golf course are located between the shoreline and a roadway that falls within a future administrative hazard zone, in which case hardening is assumed along the entire length of the shoreline fronting the golf course greens, following observed trends of hardening at golf courses			

Mosaics of shoreline imagery from 2011 to 2015, also used in the calculation of future shoreline position, are used as the baseline scenario to identify modern development.

From this recent imagery, the locations of existing shoreline hardening were digitized. The extent of the observed portion of shoreline hardening within each parcel is largely determined by land use category. For example, when a residential building is threatened by erosion hazards, the typical response is to harden the entire length of the parcel that faces the ocean; conversely, threatened buildings in beach parks (often restroom facilities) are often protected by hardened shorelines that only front the particular building and leave the rest of the parcel unhardened. These observed patterns are used to estimate future hardening for buildings and transportation under each land use category (Table 1). While actual future shoreline hardening patterns may differ, general patterns of hardening are assumed to estimate a likely scenario. Land use categories and hardened shorelines were identified to the parcel level and overlain with the administrative hazard zones. At these locations, a database was developed of alongshore length of at-risk parcels, land use category, presence or absence of modern hardening and the physical dimensions of the modern beach. The length of shoreline was measured for the following scenarios: “Hardened 2011–2015” (baseline, hardened), “At-risk 2011–2015″ (baseline, at-risk),” “At-risk 0.25 m,” “At-risk 0.46 m,” “At-risk 0.74 m,” and “Not At-risk” (see Fig. 3).

## Results

All sandy shoreline present in the early twentieth century, approximately 108 km or 60% of the entire Oʻahu shoreline, was analyzed^40^. Average shoreline change rates were considered for the island by region (Table 2). Today, over half of the shoreline is eroding (negative shoreline-change rates indicate erosion). The average shoreline change rate for the entire island is − 0.03 ± 0.01 m/year. The eastern shoreline is the only region that has a positive average rate, indicating accretion; this result reflects significant beach lengths that have continued to accumulate sand as a result of unique geologic conditions even during a century of SLR^41^. Along other portions of the east side, overdevelopment and inappropriate road placement have led to considerable shoreline hardening and beach loss. Romine et al.^26^ provide an analysis of site-specific conditions associated with both accretion and erosion along this coast.

**Table 2:** Average shoreline change rates of the sandy shorelines for the island and by region for the modern shoreline and SLR scenarios (0.25 m, 0.46 m, 0.74 m).

Region	SLR scenario	Shoreline change rate (m/year)	Percent eroding (%)	Percent accreting (%)
North	Modern	− 0.07 ± 0.03	72	28
	0.25 m	− 0.20 ± 0.05	92	8
	0.46 m	− 0.25 ± 0.06	95	5
	0.74 m	− 0.30 ± 0.08	97	3
East	Modern	0.04 ± 0.03	51	49
	0.25 m	− 0.09 ± 0.03	72	28
	0.46 m	− 0.14 ± 0.05	78	22
	0.74 m	− 0.18 ± 0.07	82	18
South	Modern	− 0.03 ± 0.01	52	48
	0.25 m	− 0.14 ± 0.03	75	25
	0.46 m	− 0.19 ± 0.05	81	19
	0.74 m	− 0.23 ± 0.07	86	14
West	Modern	− 0.20 ± 0.04	89	11
	0.25 m	− 0.33 ± 0.04	97	3
	0.46 m	− 0.39 ± 0.04	98	2
	0.74 m	− 0.44 ± 0.04	98	2
All	Modern	− 0.03 ± 0.01	61	39
	0.25 m	− 0.16 ± 0.01	80	20
	0.46 m	− 0.21 ± 0.01	85	15
	0.74 m	− 0.25 ± 0.02	89	12

By 0.25 m of SLR, every side of the island establishes a pattern of chronic erosion with the percent of eroding shoreline increasing, and the average rate of shoreline change becomes more erosive. Island-wide, under the highest SLR scenario of 0.74 m of SLR, the average percent of eroding shoreline is 89%. The most erosive segments of the island are on the west and north-facing shores, where nearly all portions of these two regions are experiencing chronic erosion with average rates of − 0.44 ± 0.04 m/year and − 0.30 ± 0.08 m/year, respectively.

**Figure 4:**
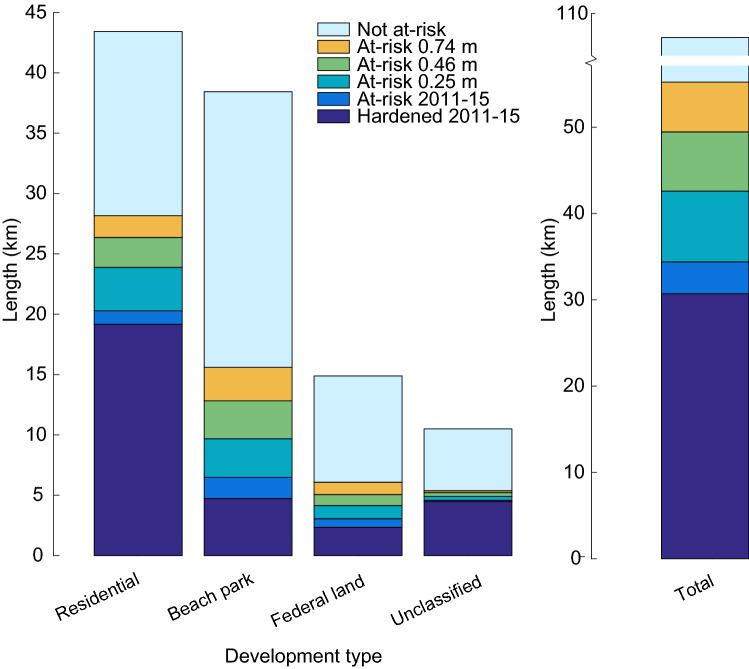
Patterns of coastal development. Length (km) of total sandy shoreline related to modern backshore land use; modern-day hardened shoreline; projected risk of hardening under modern-day and future SLR scenarios: 0.25 m, 0.46 m, 0.74 m, and totals.

**Figure 5:**
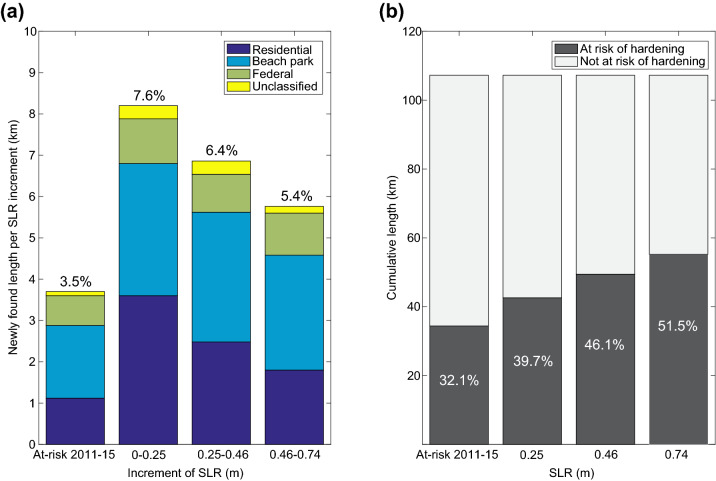
(**a**) For each increment of SLR, the length of shoreline that is newly found to be at risk of hardening is shown, with land use categories displayed in different colors. Percent values are the percent of all O‘ahu sandy shorelines that are newly found to be at risk of hardening during each SLR increment. (**b**) The cumulative length of shoreline that is either currently hardened or at risk of hardening continues to increase as sea level rises. Cumulative shoreline length is also given as a percent of all sandy shorelines on O‘ahu.

The results of intersecting backshore characteristics with the erosion hazard zones reveal patterns of modern-day backshore land use on the sandy shorelines on Oʻahu (Figs. 4, 5). Of the shoreline analyzed, modern-day backshore land use is predominantly residential (40.5%) and government-owned beach parks (35.8%) (Fig. 4). The remaining categories (federal land and unclassified areas) together constitute approximately 23.7% of backshore land use. It is found that more than one-quarter of all Oʻahu’s sandy shoreline is presently hardened, mainly residential parcels.

In addition to 28.6% of sandy shoreline that is currently hardened, GIS analysis indicates another 3.5% of O‘ahu’s sandy shorelines are currently at risk of hardening when considering the 20 ft criteria for triggering an emergency permit. At greatest risk are beach parks (1.6%) followed by residential lands (1.0%), while federal lands (0.7%) and unclassified land (0.1%) each constitute less than 1% of backshore land use at risk of new hardening.

Model results indicate that, in addition to the 32.1% of sandy shoreline that is already hardened or at risk of hardening in the modern-day scenario, an additional 7.6% of O‘ahu’s sandy shoreline will be at risk of hardening under the 0.25 m of SLR scenario (very likely by the year 2050^42^). Most of this additional at-risk area is associated with residential lands (3.4%) and with beach parks (3.0%). This increase between modern-day and 0.25 m of SLR scenario (7.6%) constitutes the largest increase of hardening risk between any of the consecutive SLR increments in this analysis (Figs. 4, 5a).

While the total amount of hardened and at risk of hardening shorefront residential lands continues to increase between 0.25 and 0.46 m of SLR (Figs. 4, 5b), the increase is less than that over the previous SLR increment (modern-day to 0.25 m of SLR) for each land use category. Notably, residential uses had the largest decrease with 2.3% newly at-risk residential use compared to the increase between the modern-day and 0.25 m SLR scenarios (3.4% newly at-risk residential land) (Figs. 4, 5a). Beach parks have the largest increase (2.9% newly at-risk land) in this SLR increment.

Results indicate that, with 0.74 m of SLR, approximately 51.5% of all sandy shorelines on Oʻahu will be hardened or at risk of hardening (Figs. 4, 5b). This constitutes an 80% increase compared to the amount of presently hardened shorelines. The highest SLR scenario triggers continued additional land at risk of hardening among all four land use categories. Newly identified (between 0.46 and 0.74 m of SLR) at-risk residential lands (1.7%), beach parks (2.6%) and unclassified lands (0.1%) indicate a slight reduction in the rate of new risk compared to lower SLR intervals, while federal land shows constant risk (Figs. 4, 5a).

## Discussion

Building on previous research of shoreline hardening patterns^18^, this report analyzes the relationship of backshore land use and SLR on shoreline hardening. Future shoreline position and rate of change are projected under three SLR scenarios (0.25, 0.46, and 0.74 m). Applying the 20 ft buffer rule^34^ currently in use by shoreline managers, four backshore land use categories are identified that have historically been protected by hardening as a policy choice and which qualify for emergency permitting under each scenario, a proxy for risk of hardening. This study shows that SLR will likely increase shoreline erosion and, after 0.74 m of SLR, put a total of 51.5% of the beaches on the island at risk of hardening because of the nature of backshore land use. Consequently, more shoreline is expected to become vulnerable to beach loss^1,4–8^, decreasing beach resources available for social, ecological, and economical uses. Unless proactive, collaborative, and conservation-oriented governance is developed, shoreline hardening and beach loss will continue to characterize coasts where backshore land use is marked by developed assets.

Results confirm the findings of Summer et al.^5^ that shoreline hardening has been used as the primary policy tool in response to chronic erosion. A total of 28.6% of all present-day sandy shoreline is already hardened, with another 3.5% found to be currently at risk of hardening. Data reveal that with only another 0.25 m of SLR, over 10% of still existing non-hardened beaches are at risk of being lost. When considering: 1) the amount of already hardened shoreline; 2) the current shoreline at risk of hardening because of assets located within 20 ft of active erosion; and 3) the model projections for hardening risk under 0.25 m of SLR, nearly 40% of beaches on Oʻahu face near-term critical management decisions that will determine their immediate conservation.

According to Sweet et al.^42^ relative to the year 2000, global mean sea level is very likely (90–100% probability) to rise 0.15–0.38 m by 2050. Consequently, management decisions regarding beach conservation made today and in the next three decades will determine the continued existence of a significant portion of the beaches on Oʻahu and other coastlines in the world with similar SLR and development patterns. It is critically important that options are developed soon for beachfront landowners and resource managers to avoid further inappropriate management decisions.

The north and west shores are identified as experiencing the greatest rates of shoreline change and percentage of eroding coast under all SLR scenarios. By midcentury (0.25 m), 92% of the north region is chronically eroding with an average rate of 0.2 ± 0.05 m/year (Table 1). In the west region, 97% of the shoreline is chronically eroding with an average rate of 0.33 ± 0.04 m/year. Consistent with the island-wide results, residential backshore land use is exposed to the greatest risk on the north shore, while beach parks have the greatest risk exposure in the west region (see Supplementary Fig. S1). These two segments are also exposed to the greatest wave energy, and this suggests the greatest risk of hardening is related to shorelines exposed to the greatest wave energy.

Beaches are large systems that work beyond land use categories, yet, are often managed on a parcel level^5^. Beachfront residential areas highlight the challenge of managing sandy shorelines because of the many private landowners involved who currently have rights to harden their shoreline for their resources, while affecting other stakeholders and shorelines beyond their property. In this analysis, the primary land use category driving risk of beach loss is residential use. By the end of the century, more than half of the residential shoreline could be hardened or at risk of hardening, which is more than a quarter of all sandy shorelines on Oʻahu.

These results likely are conservative since the common phenomena of flanking^5^, the chain reaction of shoreline hardening due to increased erosion at the ends of a hard structure, is not included in the methodology. Since most of the shoreline is residential, policies for those land use types should be addressed first followed by beach park, federal land, and unclassified land. Management of federal lands, the land use with the least clear pattern for shoreline hardening, may be improved from the patterns shown in the other land use types.

The main trigger for hardening in beach park and unclassified shoreline land use types is transportation assets. Beach park and unclassified shorelines experiencing chronic erosion will likely be allowed to recede until hardening for protection of the transportation assets is triggered, causing beach loss. This result suggests, again, that the critical time for beach conservation decisions is now and in the immediate future. A report published for Hawaiʻi’s Department of Transportation analyzed the potential effect of SLR on coastal highways and identified priority areas and remediation options, many of which were to harden or relocate roads where possible depending on land ownership, public acceptance, and costs^43^. Statements by officials about the need to discuss the future of communities to help with determining the remediation options^44^ highlights the impact of shoreline hardening beyond the development and community at the shoreline.

Interagency collaborations and public–private partnerships have not been deeply explored as avenues of beach conservation on Oʻahu or statewide. However, these relationships are necessary because of the many stakeholders along the shore as demonstrated in this study. Research on collaborative management of resources with community participation should be explored for best practices^45,46^. Because beaches are held in a public trust in Hawaiʻi^29^, stakeholders not only include those who own the beach front land, which is outlined in this study, but also the public. The public is connected to the shoreline for recreational, social, and cultural reasons. Additionally, beaches are critical ecosystems tied seaward to nearshore reefs and landward to the indigenous and endemic-rich ecologies of coastal dunes. As erosional stress builds with SLR, collaboration will be key to successful management of sandy shorelines.

As identified by Summers et al.^5^ shoreline management is largely a reactive, parcel by parcel, system of choices in Hawaiʻi that diminishes the role of proactive and place-based decisions. This study demonstrates the complexity of simultaneously managing sandy shorelines as critical environments and as sites for infrastructure deployment and investment. This study is also an example of recognizing that the past is no longer a valid guide to the future, and that analyzing entrenched practices through the lens of science can provide new planning insights before it is too late to apply them. So-called strategic retreat from the shore offers an opportunity for sandy beach survival as well as community revitalization^47^, but will likely come with financial^48^ and social challenges^49^. However, action is necessary and likely less costly than inaction even with high uncertainties^48^. Retreat may not be appropriate or possible for every shoreline, and alternatives based on local parameters should be considered. In the case of sandy beach conservation, poor shoreline hardening management practices have threatened beaches around the world historically and today and will continue if not changed. Data indicate the critical time for resolving this problem is now.

## Conclusions

- Future shoreline projections under SLR scenarios, when analyzed in terms of backshore land use, provide a globally relevant warning to resource managers and stakeholders that modern-day and immediate near-term decisions strongly impact beach conservation.
- Modeling reveals that the maximum risk of shoreline hardening, and, by extension beach loss, peaks before mid-century.
- Residential lands, beach parks, federal lands, and unclassified lands (respectively) are in critical need of new management options focused on beach conservation.
- The reactive and piecemeal approach to beach management has failed under historic policies. A new regime of decision-making that emphasizes proactive, place-based and collaborative partnerships is urgently needed if beaches are to be conserved for future generations, cultural practices, critical ecosystems, and state economies.

## Supplementary information

Supplementary information

## Data Availability

The data used in this study is available from the groups in the text or in citations. Other intermediate products are available upon request to kdat@hawaii.edu.
