# Supplementary material for: Risk of shoreline hardening and associated beach loss peaks before mid-century: Oʻahu, Hawaiʻi
Source: Sci Rep. 2020 Aug 12;10:13633. doi: 10.1038/s41598-020-70577-y (PMC7424522; doi:10.1038/s41598-020-70577-y)
Supplement: Supplementary file 1 — Supplementary information [file 41598_2020_70577_MOESM1_ESM.pdf]

Risk of shoreline hardening and associated beach loss peaks before mid-century: O‘ahu, Hawai‘i

Kammie-Dominique Tavares[a]*, Charles H. Fletcher[a], and Tiffany R. Anderson[a]

[a]Department of Earth Sciences, School of Ocean and Earth Science and Technology, University of Hawai‘i at Mānoa, 1680 East-West Rd., POST Room 721, Honolulu, Hawai‘i 96822, USA
*Kammie-Dominique Tavares; kdat@hawaii.edu

Supplementary Information

Supplementary Table S1. Files used to determine land use from the Hawaiʻi GIS Statewide Program[39].

Name of file	Last updated
Parks	September 2019
Federal Owned Land	November 2019
Parcels-TMK Zone	April 2018

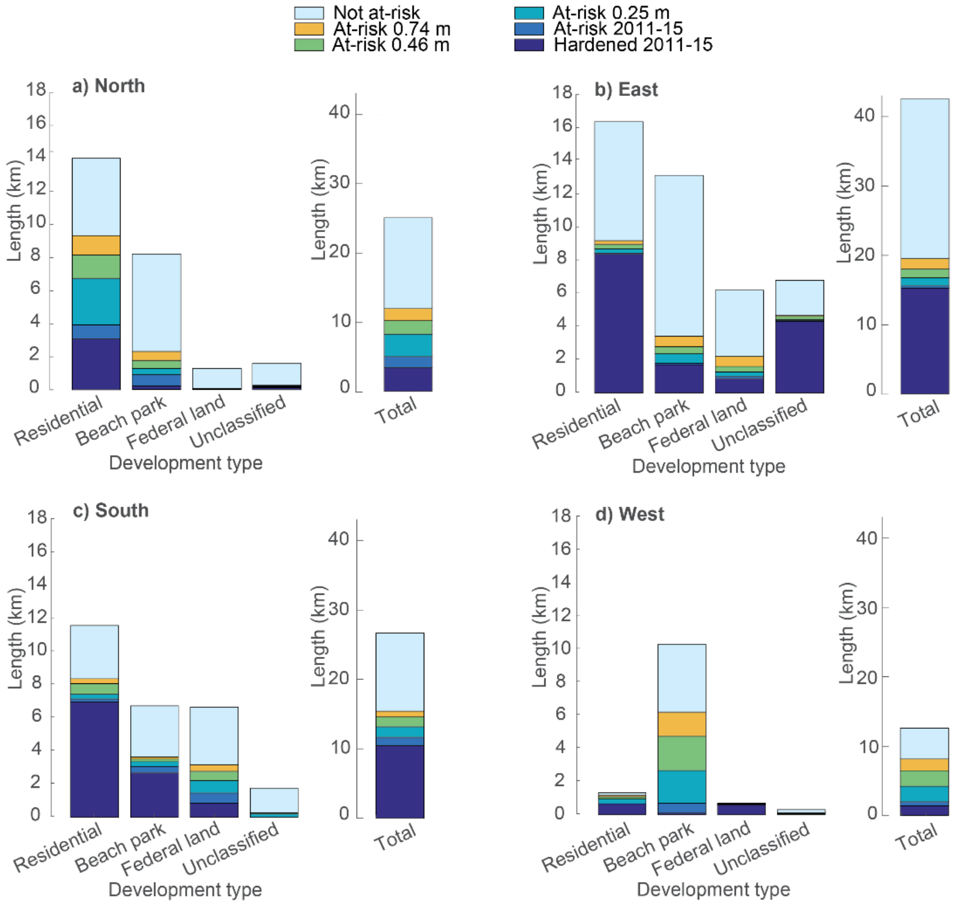

Supplementary Figure S1. Patterns of coastal development for each side of the island (a) North b) East c) South, and d) West. Length (km) of total sandy shoreline related to modern backshore land use; modern-day hardened shoreline; projected risk of hardening under modern-day and future SLR scenarios: 0.25 m, 0.46 m, 0.74 m, and totals.

Supplementary Methods

Following the method described in Anderson et al.[13] (2015), the equation for the projected vegetation line (administrative shoreline) is

$$y_{veg}(t) = y_{veg}(t_0) + r(t - t_0) - (S_f - S_{hist}) / \tan\beta$$

where $y_{veg}(t)$ is the position of the vegetation line at future time t, $y_{veg}(t_0)$ is the position at initial time t_0, r is the long-term historical shoreline change rate, $(S_f - S_{hist})$ is the difference between the IPCC projected sea level and the extrapolated O‘ahu local sea level at future time t, and $\tan\beta$ is the beach slope of the submerged portion of the beach profile.

Historical shoreline positions used in this study were extracted from aerial photographs and survey charts (t-sheets) spanning the years 1910–2007[33], and augmented with a newly digitized shoreline that was extracted from 2011–2014 satellite imagery and 2015 air photos. Referencing previous orthorectified mosaics, 0.2 m resolution air photos were rubber sheeted, and 0.5 m resolution satellite imagery was adjusted using affine transformation. Residual errors for all mosaics were kept under 2 m. Total positional uncertainty was calculated by summing in quadrature seven identified sources of error. These sources include seasonal and tidal influences, t-sheet plotting as well as its high water line to low water mark conversion, beach feature digitization, and mosaic pixel size and rectification error. Please refer to Fletcher et al.[33] (2011) for data details.

Shoreline change rates were determined by fitting a straight line to the time series of historical shoreline positions using weighted linear regression. This was done at each shore-perpendicular transect, spaced 20 m apart along all sandy shorelines of O‘ahu. Rates were smoothed alongshore with a weighting scheme of 1, 3, 5, 3, 1 for each transect[50].

Beach slope was determined from cross-shore beach surveys that were repeated twice yearly for a total of six years at 35 locations around O‘ahu. Slopes were extracted from each beach profile, and the mean and standard deviation of beach slope were determined for each profile location. Slopes were interpolated using a cubic spine if more than one profile location existed along a continuous stretch of beach.

Because of the complexity of beach processes, especially with the presence of fringing reefs, several simplifying assumptions were made in the modelling approach. It is assumed that beach retreat, or advance, will continue in the future, regardless of constraining geologic morphology, and that all land is erodible. Also, changes in wave climate and storm frequency were assumed to stay the same in the future; a recent report did not find significant changes in wave characteristics under future climate[51]. The beach profile shape was also assumed to stay in an equilibrium shape, as sea level rises. It is important to note that while the probabilistic model produces a range of possible outcomes, we use only one value, corresponding to the maximum probability density, as the modeled future shoreline position which our risk assessment is based on.
